# Pathogen Stopping and Metabolism Modulation Are Key Points to *Linum usitatissimum* L. Early Response against *Fusarium oxysporum*

**DOI:** 10.3390/plants12101963

**Published:** 2023-05-12

**Authors:** Yannis Maillot, Gaëlle Mongelard, Anthony Quéro, Hervé Demailly, Stéphanie Guénin, Laurent Gutierrez, Christophe Pineau, Sylvain Lecomte, David Mathiron, Redouan Elboutachfaiti, Jean-Xavier Fontaine, Roland Molinié, Emmanuel Petit

**Affiliations:** 1 BIOPI, UMRt BioEcoAgro 1158-INRAE, Université de Picardie Jules Verne, 1 Rue des Louvels, F-80000 Amiens, France; 2 Centre de Ressources Régionales en Biologie Moléculaire, Université de Picardie Jules Verne, Bâtiment Serres-Transfert Rue Dallery, Passage du Sourire d’Avril, F-80039 Amiens, France; 3 Linéa Semences, 20 Avenue Saget, F-60210 Grandvilliers, France; 4 Plateforme Analytique, Université de Picardie Jules Verne, Bâtiment Serres-Transfert Rue Dallery, Passage du Sourire d’Avril, F-80039 Amiens, France

**Keywords:** flax, *Fusarium oxysporum*, biotic stress, phenotyping, metabolomic, qPCR

## Abstract

*Fusarium oxysporum* is the one of the most common and impactful pathogens of flax. Cultivars of flax that show resistance to this pathogen have previously been identified. To better understand the mechanisms that are responsible for this resistance, we conducted time-lapse analysis of one susceptible and one resistant cultivar over a two-week period following infection. We also monitored changes in some metabolites. The susceptible cultivar showed a strong onset of symptoms from 6 to 8 days after inoculation, which at this time point, was associated with changes in metabolites in both cultivars. The resistant cultivar maintained its height and normal photosynthetic capacity but showed a reduced growth of its secondary stems. This resistance was correlated with the containment of the pathogen at the root level, and an increase in some metabolites related to the phenylpropanoid pathway.

## 1. Introduction

*Linum usitatissimum* L., is a dicotyledonous annual crop plant cultivated for thousands of years for its fiber or for its oil [1,2]. Linen from fiber flax is used in the textile industry and oil from oilseed flax is mostly known for non-food outlets, such as linoleum manufacturing, printing inks or varnish, but also for livestock feeding. Recently, the interest in flaxseed oil for human consumption has grown notably due to its nutritional properties. Indeed, this oil mainly contains omega 3 polyunsaturated fatty acids such as alpha-linolenic acid (between 52% to 73% of the whole oil content) [3] and other nutrients such as vitamin E [4]. Moreover, flax seeds contain high levels of lignans (more than in any other crop plant) [5], which are known for their anti-inflammatory, antioxidative and antiestrogenic activities, which can prevent different types of cancer, such as colorectal, breast or prostate cancers [6,7]. Due to the numerous properties of its oil, during the last decade, the economic interest in linseed has increased, as well as its cultivated area, especially in Asia and Europe [8].

As for any crop plants of interest, flax is subjected to pathogen attacks, especially from fungi pathogens. *Fusarium oxysporum* is one of the most usual and harmful pathogens affecting flax, with an estimation of 20% of crop loss [9]. This necrotrophic soil-borne fungus colonizes plant tissues by penetrating the roots using a cocktail of enzymes such as cellulase, pectinase or protease, thus degrading the plant cell wall [10]. The pathogen is able to spread inside host tissues through the vascular system where mycelium develops into xylem vessels [11]. The consecutive vessel obstruction causes nutritional disorder by stopping the vascular flow, leading to the yellowing and death of the host plant [12,13]. In addition, this fungus causes a primary metabolism disorder, notably in the polyamine and pectin metabolism [14,15]. *Fusarium oxysporum* produces three different types of asexual spores: chlamydospores, which act as primary inoculating spores that are able to survive in the soil for a long time due to a perennial structural form; and macroconidia and microconidia, which allow the fungus to spread and infect surrounding plants after the colonization of an initial host [16]. Such capabilities provide good resistance against fungicides and allow the pathogen to contaminate soil and crop plants after a long time of dormancy. The mechanisms sustaining *Fusarium oxysporum* infection and flax responses remain poorly documented, and the implementation of indirect means, such as cultural rotation, crop burning or prevention, is therefore the only current way to fight against the pathogen.

Plants have developed many means of defense against pathogens, especially against fungi that are capable of digesting the cell wall and penetrating deeply into the organism. For effective defense, the plant must detect the aggression very early by sensing molecules that are generated during the beginning of the attack. Those molecules can be exogenous, derived from the pathogen and constitute the Pathogen-Associated Molecular Pattern (PAMP). Otherwise, they can be endogenous, produced by the plant and part of the Damage-Associated Molecular Pattern (DAMP). The first and common defense mechanism activated by the plant, which follows the detection of the attack and takes place a few hours after the infection, is the Hypersensitive Response (HR); this is associated with the programmed apoptosis of plant cells surrounding the infected zone [17]. Other defense mechanisms are triggered during this interaction phase, including the generation of Reactive Oxygen Species (ROS), which are known to be linked to the expression of different defense genes [12]. Some of these genes lead to the production of Pathogenesis-Related proteins (or PR proteins), comprising glucanase, chitinase and various enzymes that degrade the fungal cell wall, which directly react against the fungus. Other molecules associated with the secondary metabolism, such as phytoalexins, display many activities that are likely to help plants to directly fight against the pathogen. These molecules also provide resistance and prevention against some of the resulting effects of the defense establishment, like ROS accumulation, that could be noxious for the plant [18,19].

In this present study, the effects of a *Fusarium oxysporum* f. sp. *lini* infection on plant phenotype and primary metabolism modifications are described in two different flax cultivars, which display different disease susceptibilities. For each flax cultivar that displays specific phenotype and metabolic responses to the infection, the hypothesis of a link between the resistance and a chemical-based defense is discussed.

## 2. Results

### 2.1. Effect of Infection on Plant Phenotype

**Figure 1:**
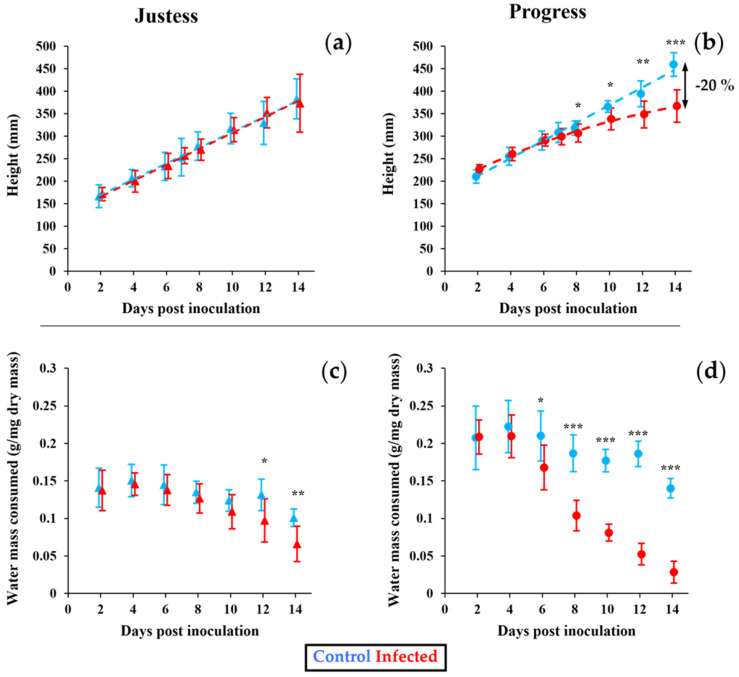
Assessment of plant height and water consumption in infected and control flax plants. Plant height measurement in infected and control plants from Justess (resistant cultivar) (**a**) and Progress (susceptible cultivar) (**b**). Water consumption in Justess (**c**) and Progress (**d**) plants assessed every two days, expressed as the loss of hydroponic medium quantity per mg of plant dry matter. Justess data are represented by triangles and Progress by circles. Significant differences between control and infected plants are indicated by asterisks. Mann–Whitney test (* for *p* < 0.05, ** for *p* < 0.01 and *** for *p* < 0.001). Error bars represent standard deviation of dataset per condition (n = 8).

In order to characterize the effects of *Fusarium oxysporum* infection in the two flax cultivars, we measured the height of the aerial part (Figure 1a,b) and the water consumption (Figure 1c,d) in plants over the course of the infection, up to 14 days after inoculation (DAI). In the resistant cultivar Justess, the height of the aerial part of the infected plants was not impacted and the infection only led to a slight reduction in water consumption, appearing from 12 DAI (Figure 1a,c). On the contrary, in plants from the susceptible cultivar Progress, infection led to a reduction in the growth height occurring from 8 DAI, the difference in height reaching up to 20% in the 14 DAI plants compared to the controls (Figure 1b). Moreover, in Progress plants, the infection triggered a strong decrease in water consumption, which turned out to be significant as soon as 6 DAI, i.e., earlier than the reduction observed in height (Figure 1d). Furthermore, the aerial part of the infected Progress plants was characterized by light yellowing and a wilted shoot tip (Supplementary Figure S1).

**Figure 2:**
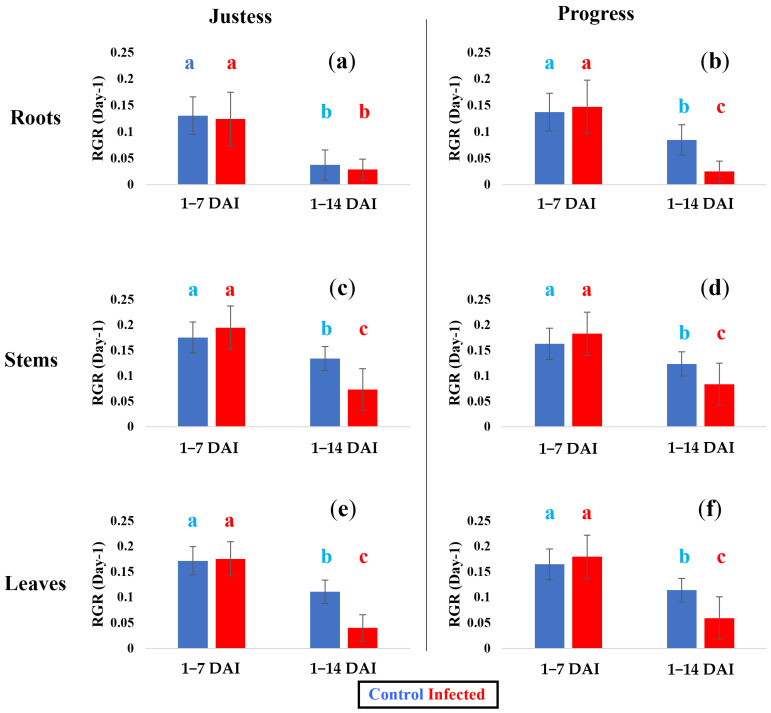
Relative Growth Rate (RGR) values obtained from dry weight in roots (**a**,**b**), stems (**c**,**d**) and leaves (**e**,**f**) in Justess (resistant cultivar) and Progress (susceptible cultivar) cultivars under control and infected conditions. Each value is calculated during two periods: from 1 to 7 days, and from 7 to 14 days after inoculation (DAI). Error bars represent standard deviation of dataset per condition (n = 8). Letters represents different statistical groups (Kruskal–Wallis test, *p* < 0.05).

In order to confirm the effect of the infection on plant growth, the plant dry mass was measured and the Relative Growth Rates (RGR) were calculated [20]. Figure 2 shows the RGR for three different organs: roots (Figure 2a,b), stems (Figure 2c,d) and leaves (Figure 2e,f). Two periods of infection were studied by calculating the RGR from 1 to 7 DAI (first week) and from 7 to 14 DAI (second week). Whatever the cultivar or organ, the infection did not lead to any significant RGR difference between the infected and control plants during the first week of infection. During the second week of infection, the RGR value turned out to be systematically lower than the one observed in the first week, which makes sense given that all the plants were in a natural decelerating phase of vegetative growth [21]. Interestingly, at this stage, the infection triggered a significant reduction of the RGR of the stems and leaves from both cultivars. The infection led to a reduction in the RGR of the Justess stem while the plant height remained unchanged; these data suggest that Justess is able to maintain a resilient ability to grow in height, even though its biomass is affected by the infection. Moreover, the infection had no effect on growth in the Justess roots.

Additional analyses were performed using the PlantScreen™ phenotyping system (Photon Systems Instruments). Visible and fluorescence imaging, using RGB and FluorCam top cameras, respectively, provided data from the aerial parts of both flax cultivars in each condition (Supplementary Figures S1 and S2). An analysis of the data obtained using fluorescence imaging allowed the efficiency of the photosystem II to be determined via the maximum quantum yield (QY max) calculation [22] (Supplementary Figure S2a,b). Interestingly, while the photosystem yield was affected in the leaves of the susceptible Progress cultivar from 8 DAI, the infection had no significant effect in the resistant Justess cultivar. Then, by analyzing the RGB images, the relative quantification of the plant area belonging to each of the three different color groups, green, yellow-green and yellow, was performed on the aerial parts of the plants from 2 to 14 DAI. Over the course of the infection, the resistant Justess cultivar displayed stable ratios for pixels belonging to each of the three groups (Supplementary Figure S2c,e,g), indicating a stable color in the aerial part that was consistent with the stable photosystem yield previously measured. On the contrary, in the susceptible Progress cultivar, color modification was revealed from 8 DAI, characterized by a yellowing, i.e., a decrease in “green” pixel ratio and an increase in the “yellow-green” and “yellow” pixel ratios (Supplementary Figure S2d,f,h). This color change was likely related to the photosystem yield modification that was quantified in this cultivar.

Altogether, our phenotyping results indicate that, while the infection led to strong physiological impairments in the susceptible flax cultivar, the resistant flax cultivar appeared to be much less affected.

### 2.2. Dynamics of Pathogen Levels in Plant

**Figure 3:**
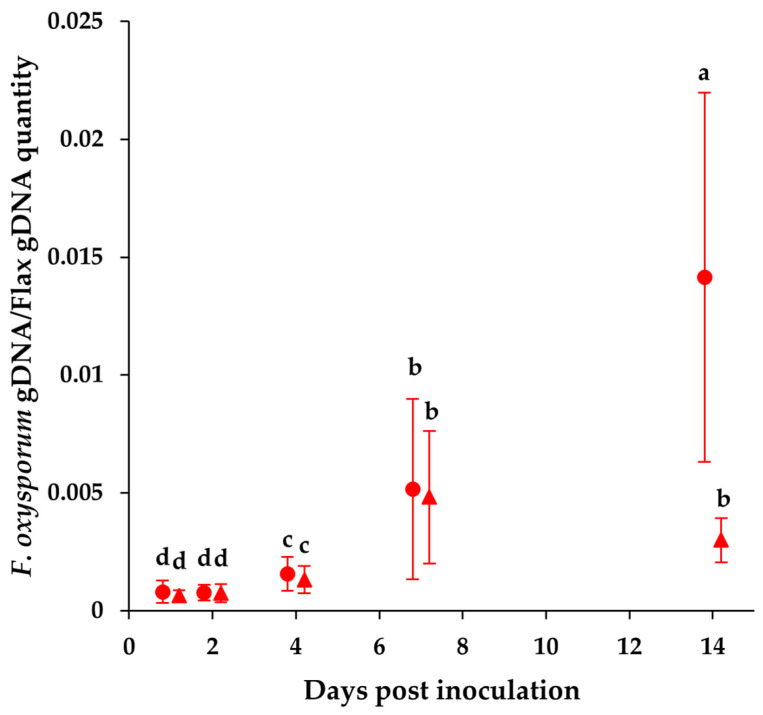
Pathogen DNA (*Fusarium oxysporum*) relative quantity in Justess (resistant cultivar) (triangle) and Progress (susceptible cultivar) (circle) roots measured using qPCR. Error bars represent the standard deviation of the dataset per condition (n = 8). Significant differences are marked by groups a, b, c and d (Mann–Whitney test, *p* < 0.05).

The level of pathogen in the plant roots was followed over the course of the infection by quantifying fungal DNA using quantitative PCR (qPCR). Figure 3 represents the relative pathogen DNA quantity (quantity of *Fusarium* DNA/quantity of flax DNA) assessed under the infected condition in each cultivar at 2, 4, 7 and 14 DAI. No pathogen DNA was detected in the control plants. In infected plants, the pathogen DNA was detected from 2 DAI and its level increased by 6 times at 7 DAI in both cultivars. Interestingly, the level of pathogen DNA in the root of the resistant cultivar Justess remained the same at 14 DAI as that at 7 DAI, whereas it increased by 4 times in Progress roots. In order to analyze the pathogen propagation within the plant tissues, the quantification of fungal DNA was performed in the other organs of the infected flax plant. However, it was only detected at a low level in Progress stems at 14 DAI (data not shown). This result indicates that *Fusarium oxysporum* reached the aerial parts of the susceptible cultivar but not those of the resistant cultivar, where it seems that the pathogen remained confined to the roots. The low level of pathogen detected in the aerial part in Progress, as well as its confinement to the roots in Justess, means that both cultivars displayed strategies that aimed to restrain pathogen propagation. However, a different or higher response was implemented in the resistant cultivar, which turned out to be more efficient. This response could take different forms, and, for a better characterization, the symptomatic effects of the disease were investigated at the metabolomic level, especially around 7 DAI, which appears to be a key time for the plant response.

### 2.3. Effect of Infection on Organic and Inorganic Solute Contents in Flax Leaf

To better understand the effect of *Fusarium oxysporum* on flax physiology, the content of some of the compounds involved in primary metabolism was monitored; GC–MS analysis was used for primary metabolites and HPLC analysis was used for inorganic solutes. A total of 46 compounds were found, including 17 amino acids and derivatives, 14 non-structural carbohydrates, 7 organic acids, 4 inorganic anions and 4 inorganic cations.

**Figure 4:**
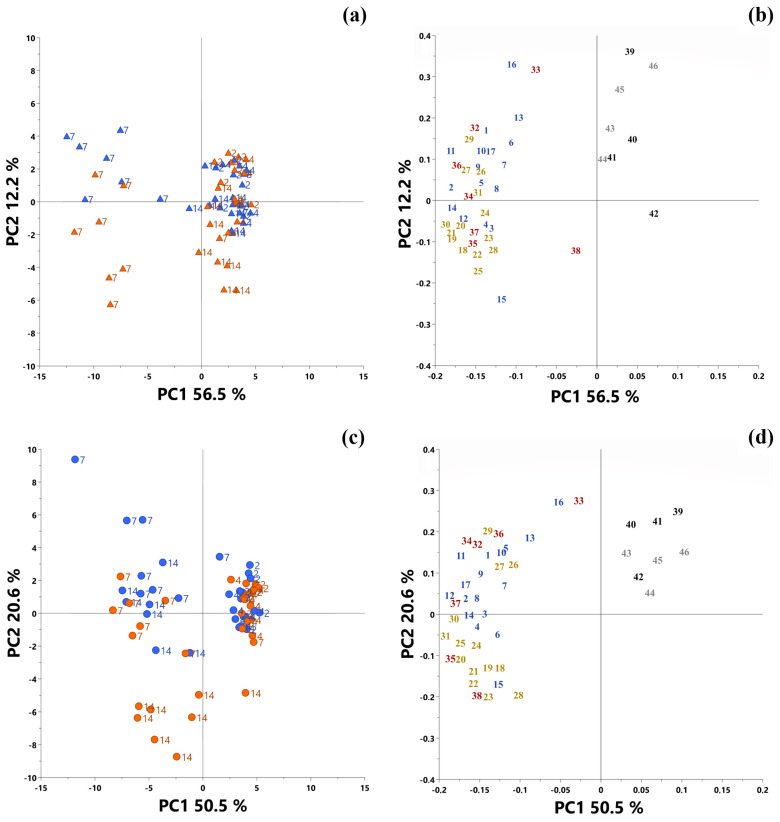
Principal component analysis of targeted metabolites quantified in infected and non-infected leaves from 2, 4, 7 and 14 days after inoculation in plants. Score plot (**a**) and loading plot (**b**) for the resistant cultivar, Justess, and score plot (**c**) and loading plot (**d**) for the susceptible cultivar, Progress. Plant individuals are represented in score plots (**a**,**c**) by colored spots: blue for control condition and red for infected condition. Each metabolite in the loading plot is represented by a colored number: inorganic anions in grey, inorganic cations in black, amino acids and derivatives in blue, non-structural carbohydrates in yellow and organic acids in red. 1: Alanine, 2: Valine, 3: Leucine, 4: Isoleucine, 5: Proline, 6: Glycine, 7: Serine, 8: Threonine, 9: Aspartate, 10: GABA, 11: Glutamate, 12: Phenylalanine, 13: Glutamine, 14: Lysine, 15: Tryptophan, 16: Putrescine, 17: Ethanolamine, 18: Xylose, 19: Arabinose, 20: Rhamnose, 21: Fucose, 22: Fructose, 23: Sorbose, 24: Glucose, 25: Myo-inositol, 26: Fructose-6-phosphate, 27: Glucose-6-phosphate, 28: Sucrose, 29: [Threonate], 30: [Erythreonate], 31: Tartarate, 32: Succinate, 33: Fumarate, 34: Malate, 35: α-ketoglutarate, 36: Citrate, 37: Quinate, 38: Chlorogenate, 39: K^+^, 40: Mg^2+^, 41: Ca^2+^, 42: Na^+^, 43: Cl^−^, 44: PO_4_^3−^, 45: SO_4_^2−^, 46: NO_3_^−^. Compounds between brackets are putatively identified by their MS fragments and not by standards.

Root extracts from both cultivars at 2, 4, 7 and 14 DAI were analyzed in control and infected conditions. The data produced were used to perform a principal component analysis in order to obtain a global view of the effect of the infection induced by *Fusarium oxysporum* on flax metabolism. In order to avoid any genotype effect, an individual PCA was performed for each cultivar. Thus, the PCA performed using the data obtained from Justess described 68.7% of the total variability, 56.5% for principal component 1 and 12.2% for principal component 2 (Figure 4a). No discrimination was observed at 2 and 4 DAI in the Justess plants. After a longer period of infection (7 and 14 DAI), a difference was observed between the control and infected plants, according to component 2. This was associated with a significant decrease in the amino acid and derivative content and a significant increase in the non-structural carbohydrates content in the infected plants (Figure 4b and Figure S3). In Progress, the PCA explained 71.1% of the total variability, 50.5% for principal component 1 and 20.6% for principal component 2 (Figure 4c). As for Justess, no difference was observed between the control and infected plants at 2 and 4 DAI. At 7 DAI, a slight difference was observed between the samples obtained from the control and infected plants, which became significant at 14 DAI in the plants. Like in Justess, this difference was characterized by component 2 and was associated with a significant decrease in the amino acid and derivative content and a significant increase in the non-structural carbohydrates content in infected plants (Figure 4d and Figure S3).

**Figure 5:**
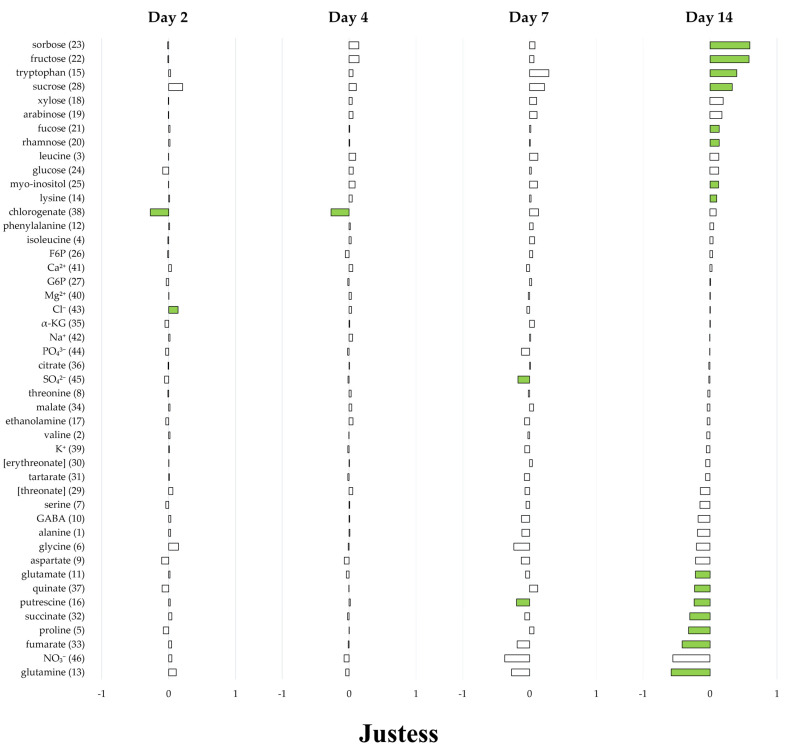

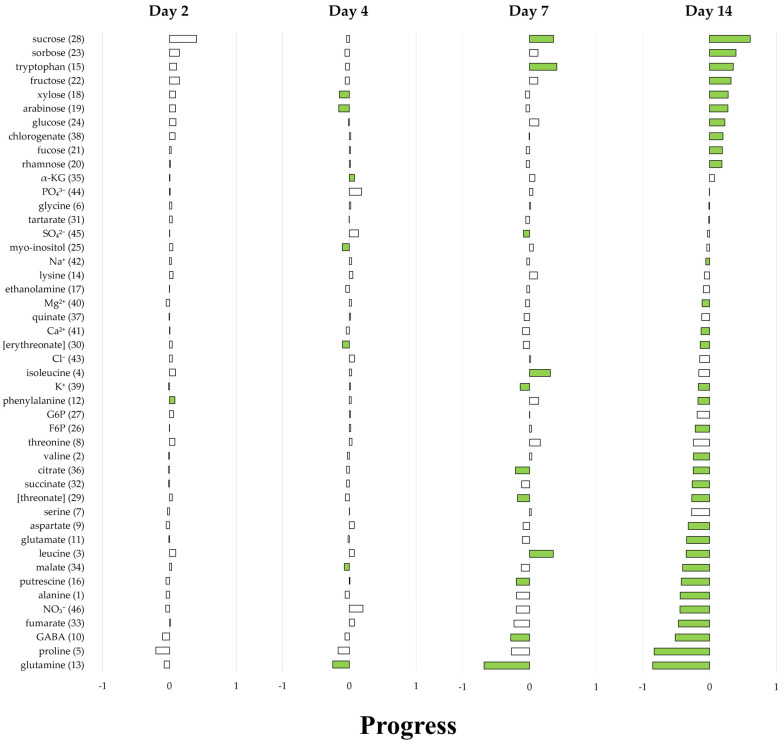
Relative amount of metabolites and inorganic ions (Infected/Control conditions, Log10) in Justess (resistant cultivar) and Progress (susceptible cultivar) leaves. Each compound is associated with a number: 1–17, amino acids and derivatives; 18–31, non-structural carbohydrates 18–31; 32–38, organic acids; 39–42, inorganic cations; 43–46, inorganic anions. Significance (*p* < 0.05) is shown by the green color (Mann–Whitney test). Compounds between brackets are putatively identified by their MS fragments and not by standards.

The data were then processed via univariate analyses in order to show the variations in each compound level during the infection kinetic (Figure 5). In Justess, at 2 DAI, only chloride and chlorogenate were differentially accumulated in infected plants compared to the controls, with a ~0.7-fold increase and ~1.9-fold decrease, respectively. This difference remained only for chlorogenate in infected plants at 4 DAI. At 7 DAI, variations were still subtle, observed only for putrescine and sulfate, whose contents saw a ~1.6-fold and ~1.5-fold decrease, respectively, in infected plants. Interestingly, a stronger modification of the metabolism was observed in infected plants at 14 DAI, where the level of 15 compounds out of 46 was modified. At this stage, the infection by *Fusarium oxysporum* led to a decrease, from ~1.6-fold to ~3.8-fold, in the contents in amino acids and derivatives, such as glutamine, proline or glutamate. Concomitantly, an increase in the levels of non-structural carbohydrates, such as fructose, sucrose, and sorbose, was observed, ranging from ~2.1-fold to ~4-fold.

In Progress, the first metabolic changes occurred in infected plants from 4 DAI, consisting of a small decrease in the contents of xylose, arabinose, or glutamine, ranging from 1.4-fold to 1.8-fold. At 7 DAI, infected plants displayed stronger metabolic changes, mainly for amino acids and derivatives, and particularly for glutamine (~4.8-fold decreased level) and tryptophane (~2.6-fold increased level), as well as for sucrose (~2.3-fold increase). At 14 DAI, the infection triggered a wide modification of the metabolism, affecting the content of almost all the studied compounds. This disruption resulted in a relatively strong decrease in the content of amino acid compounds and derivatives, such as glutamine or proline (~7-fold and ~6.7-fold, respectively), as well as an opposite increase in the level of non-structural carbohydrates, mainly sorbose, fructose and sucrose (~2.5-fold, ~2.1-fold and~4-fold, respectively). Interestingly, among these non-structural carbohydrates, a slight increase in the levels of rare sugars (~1.4-fold), including fucose and rhamnose, was quantified. In addition, it is noteworthy that the level of tryptophan saw a ~2.5-fold increase in infected plants at 14 DAI, whereas the infection reduced the levels of the other amino acids.

## 3. Discussion

### 3.1. Effects of the Fusariose Disease on Flax Plant

#### 3.1.1. Growth Retardation and Energetic Reconfiguration

**Figure 6:**
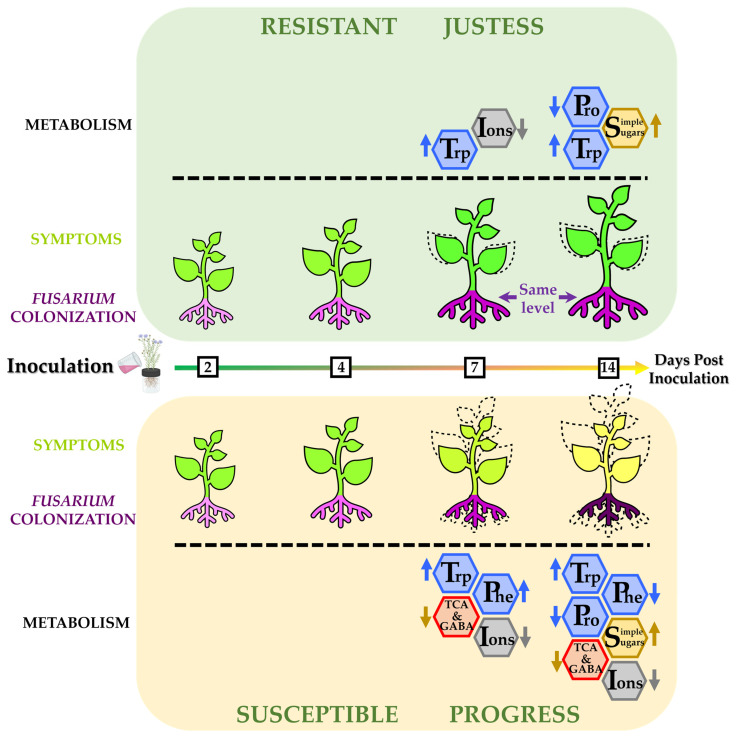
Timeline of symptom apparition, pathogen colonization and primary metabolome modulation during *Fusarium oxysporum* infection, from inoculation up to 2 weeks later, in Justess and Progress flax cultivars. Darkening of root color indicates a higher concentration in fungal gDNA. Arrows represent an increased or decreased concentration of a metabolite or a group of metabolites compared to the control condition.

The phenotypic data show the strong impact of the disease caused by *Fusarium oxysporum* infection on the susceptible flax cultivar, which displays much more severe symptoms than the resistant one (summary in Figure 6).

The experimental modalities we used to follow up on the disease were similar to the ones that were set up by Chen et al. (2020) [23] on the model *Fusarium fujikuroi* rice, where a similar pattern of pathogen colonization was shown through the diseased plants. Indeed, in this previous study, the pathogenic agent was detected mainly in roots and at the base of stems; this is similar to our present results, where little or no fungal DNA was detected in the leaves of infected plants. In addition, both resistant and susceptible cultivars of rice were shown to be infected and their roots were similarly colonized after 14 days of infection. But after 21 days, the amount of pathogen dramatically increased, especially in the susceptible cultivar.

The colonization pattern of *Fusarium fujikuroi*, as well as the behavior of the susceptible and resistant rice cultivars during the infection, are very comparable to what we show in the present study with the *Fusarium oxysporum* flax model. In our case, the pathogen level increases drastically in the root of the susceptible cultivar at 14 days after inoculation, whereas this level remains low in the resistant cultivar (Figure 3). This suggests that keeping a stable and low level of pathogen in the root over the course of the infection constitutes an effective defense mechanism that offers better resistance to the plant. This hypothesis makes sense, since limiting the quantity of pathogen in the root could reduce its development within xylem vessels, thereby allowing the maintenance of a normal sap uptake, as suggested by the stable water consumption in the resistant cultivar (Figure 1). This is likely to lead to stable photosynthesis activity and the steady growth in height of the aerial parts of the infected resistant plant (Figure 1 and Figure S2).

It is interesting to notice that in control conditions, the resistant cultivar Justess is shorter than the susceptible cultivar Progress. Indeed, the average height of Justess plants is ~50 mm lower than that of Progress plants during the whole developmental kinetic studied in this work (Figure 1a,b). A lower height is a common feature of plants presenting a resistance against biotic stresses [23]. Such a phenotype is often associated with the better ability of a plant to face pathogen attacks via primary metabolism remodeling [24]. In the same way, we also observe a lower relative growth rate (RGR) in the roots of the resistant cultivar Justess compared to Progress in the control condition (Figure 2a,b). Interestingly, while the root is the entry point of the infection, the growth of Justess roots is dramatically slow even if the pathogen level is contained at a low level in these roots. This reinforces the possibility of a link between a better resistance to *Fusarium* and the slow growth of flax plants, which is likely to disadvantage pathogen propagation through the plant tissues. This hypothesis obviously needs to be confirmed in further studies, but it already constitutes an interesting avenue to be explored in flax breeding.

Regarding the stem development, although the resistant cultivar Justess did not display any height growth retardation during infection (Figure 1), at the same time, a decrease in the RGR was measured for its aerial part (Figure 2), which indicated a lower biomass production. This counterintuitive effect can be explained by looking at the plant architecture. In reaction to the disease, the secondary stems of Justess were subject to growth retardation, while its main stem was not affected (Supplementary Figure S1). Therefore, the decrease in the RGR of the aerial part of Justess was only related to the reduction in biomass production from the secondary stems. On the contrary, in the infected Progress plants, the decrease in the RGR of the aerial part was clearly due to growth retardation in the main and in the secondary stems, which were both affected similarly by the infection. Therefore, it appears that these secondary stems are more affected by the *Fusarium* infection.

The yellowing and the reduction in the photosynthesis efficiency displayed by the susceptible Progress cultivar (Supplementary Figure S2) are in accordance with the fact that this is a classical reaction against biotic stresses in plants, notably in flax [15]. Indeed, the photosynthesis reduction could be a strategy to remobilize energetic resources into the plant defenses, and even reduce oxidative damage. Moreover, the coordination of the carbon and nitrogen supply is known to be associated with the level of sugars in leaves [25,26]. Yet, a relative sugars accumulation is visible in our data (as shown in Figure 5 and Figure S3) and could be linked to the plant yellowing and loss of photosynthetic capacity (Supplementary Figure S2), even if the effect is barely visible in the resistant variety.

#### 3.1.2. Hydric Disturbance without Classical Abiotic Stresses Characteristics

It has been known for years that the *Fusarium* pathogen spreads inside host tissues via the development of mycelium into xylem, producing vessel obstruction that affects the vascular flow, finally leading to nutritional disorder that causes the yellowing of the plant [11,12,13]. Based on these previous studies and considering the reduction in water consumption observed in the infected Progress plant (Figure 1), an alternative hypothesis might be that the yellowing and the reduction in photosynthesis efficiency could simply result from the reduced sap uptake due to the pathogen development within the xylem vessel. The lower mineral ions level measured in infected Progress plants seems to sustain this hypothesis. Indeed, such inorganic solutes are known to be directly involved in osmoregulation, signalling or nutrient storage in plants [27].

The relative concentration of amino acids, which contribute mainly to dynamism in the PCA (Figure 4), rose during the infection in both cultivars. However, the relative quantity of γ-amino butyric acid (GABA), a non-protein amino acid known to be accumulated in abiotic stress conditions [28,29], decreased after one week of infection in the infected leaves of Progress (no significant decrease in Justess leaves) (Figure 5 and Figure S3). The same pattern was observed for proline, whose level decreased during infection in both cultivars but to a lesser extent in Justess leaves; indeed, proline is known to be highly accumulated in flax during osmotic stress [30]. Therefore, the reduced levels of these two major markers during infection, despite the strong reduction in water consumption in Progress, suggest that the infected plants from both cultivars were not exposed to hydric stress. Therefore, it turns out that the response of the aerial part of the plant to the infection is not likely to be the consequence of abiotic stress, but rather to a specific biotic stress triggered by the pathogen at the root level.

### 3.2. Resistant versus Susceptible Cultivar: Clue of Defence Mechanisms

The phenotypic and qPCR data show clear differences in the plant reaction against the disease between both studied cultivars. However, the modulation of their primary metabolism in response to the infection remains very similar. Our work provides the first metabolomic study during *Fusarium* infection of two flax cultivars displaying different levels of pathogen sensitivity. At this point, it is not possible to conclude that Justess implements a specific mechanism that explains its higher resistance.

On the other hand, the basal and late-induced modifications to the metabolic content are different in the resistant Justess flax cultivar compared to Progress, and coincide with the differential level of *F. oxysporum* colonization between both cultivars (Figure 3); this highlights the possibility of specific defense strategies being used by Justess. Indeed, in the control condition, some compounds show a basal level that is lower in Justess. For example, non-structural carbohydrates, such as sorbose or fructose, respectively, show an average level that is 2.4-fold and 2.3-fold lower in Justess than in Progress control plants. Concerning amino acids, only tryptophan displays an average basal level that is 2.3-fold lower in Justess compared to Progress (Supplementary Figure S3).

The lower level of tryptophan may be interpreted as a better conversion of this base peptide into protein, as well as some others such as valine and glutamate, which show a similar content evolution (Supplementary Figure S3). The rapid generation of proteins could confer a better response to Justess.

Phenylalanine, another aromatic amino acid, shows a strong increase at 7 DAI in both control cultivars, which is more important in the resistant Justess cultivar (Supplementary Figure S3). During the first week, the levels of phenylalanine are identical in both cultivars, regardless of the infection. However, the rise in the levels of this compound in Justess is 1.43-fold higher than in Progress in the control condition at 7 DAI. This higher concentration shown in the Justess plant during its development could be involved in the improvement in its resistance to *Fusarium oxysporum*. Indeed, phenylalanine is at the start of the phenylpropanoid pathway, a complex category of plant secondary metabolites that regroup a large diversity of polyphenols, such as lignans, hydroxycinnamic acids or flavonoids. These phenolic compounds are very well known to have strong biological properties, which are especially involved in plant defense against pathogens. They include antioxidant molecules, which help the plant to face oxidative burst, or antifungal compounds that can have a direct impact on the pathogen [9].

## 4. Materials and Methods

### 4.1. Cultivar Choice, Pathosystem Conditions and Sampling

*Linum usitatissimum* (flax seed) cultivars “Progress” (spring oilseed flax registered in 2016) and “Justess” (spring oilseed flax registered in 2020) were provided by Linéa Semences (flax breeder company). They were selected for their contrasted sensitivity to the disease, Progress being more susceptible than Justess, and for their high genealogic proximity, sharing 25% of their genetic heritage according to the breeder’s data.

These cultivars were cultivated using a hydroponic method in order to easily induce homogeneous infection via the roots, with the use of a constant amount of pathogen. Infection was performed on two-week-old flax seedlings from each cultivar, by inoculating 10^5^ microconidia/mL into the growing medium. The control and infected plant leaves, stems and roots were sampled at 2, 4, 7 and 14 days post inoculation to perform growth rate and DNA quantification via qPCR and metabolic analyses.

In parallel, plant phenotyping was monitored every 2 days during the infection. The plant and culture medium weights were assessed over the course of infection in order to deduce the kinetics of plant water consumption. Pictures of plants were automatically taken with visible and fluorescence cameras (see materials and methods), whose analysis provided morphometric and physiological data, such as height, yellowing and photosynthetic capacity.

### 4.2. Cultural Method

Before use, seeds were sterilized for 10 min in ethanol 70%, rinsed for 10 min in ethanol 96% and then dried in petri dishes under a laminar flow hood. Next, 20 seeds were deposed on the surface of 1.1% agar in petri dishes placed for 3 days at 4 °C in the dark; these were then incubated at 21 °C in a dark condition for 2 days. When the radicles reached 0.5 to 1 cm in length, each seedling was placed into a 0.5 mL microtube filled with 0.6% agar and cut at its base. Radicles were then acclimated for 2 days in the following culture conditions: 21 °C, 120 µE·m^2^·s^−1^, 16 h/day and 100% humidity. Seedlings were transferred in 0.5× Hoagland hydroponic medium into 200 mL capacity jars (2 plants per jar, each jar being considered as 1 biological replicate). Hydroponic media were renewed every two days until the end of the experiment. For this study, a total of 160 individuals were grown and divided into the different conditions (2 cultivars, control/infected, 5 harvest times and 8 biological replicates per condition).

### 4.3. Fungal Liquid Culture

*Fusarium oxysporum* f. sp. *lini*, isolated from symptomatic infected flax, was cultivated on potato dextrose agar solid medium for 7 days before the addition of 7 mL of sterile deionized water in order to suspend the microconidia. Then, 1 mL of this suspension was collected, diluted 10 times, and used to inoculate 400 mL of 24 g/L potato dextrose broth liquid medium in a 2 L flask. After 4 days of culture at 21 °C in the dark, the microconidia concentration was determined using a hemacytometer and adjusted to obtain an inoculum of 10^5^ microconidia/mL using plant culture medium.

### 4.4. Plant Inoculation

After two weeks of hydroponic culture, the plant roots were soaked in the 10^5^ microconidia/mL fungal inoculum for 4 h and then retransferred in a fresh Hoagland medium.

### 4.5. Phenotyping

Two weeks after inoculation, the whole flaxseeds aerial parts were phenotyped in a PlantScreen™ system (Photon System Instruments (PSI), Drasov, Czech republic).

1. Height Measurements: Side-view RGB pictures were automatically taken to measure plant heights.
2. Color classification: Color groups were defined using 19 different colors matching the coloring of the flax. The digital image processing method was conducted according to Bai et al. (2018) [31]: every RGB code of 19 pixel colors was converted to a Hue Saturation Value (HSV) color space and gathered into 3 color classes. The “green” class gathers Hue values between 81° and 140°, the “yellow-green” class between 61° and 80°, and the “yellow” class between 51° and 60°.
3. Photosynthetic measurements: The Fo and Fm were measured on 20 min dark-adapted plants using a FluorCam FC-800MF Pulse-Amplitude-Modulated (PAM) system manufactured by the PSI company. The maximum quantum yield of the photosystem II (QYmax) was subsequently calculated by the PlantScreen™ DataAnalyzer software. Fluorescence pictures were taken from the top of the plants.
4. Water consumption: Water consumption was determined by weighing the hydroponic pots before and after each medium renewal. Values were normalized to the dry weight obtained after the freeze-drying of each plant.

### 4.6. Growth Rate Measurement and Calculation

Every collected and ground organ was weighed using a precision scale (±0.1 mg). The relative growth rate (RGR) was determined using the following formula:

(1) $$RGR=\frac{ln\;w_{2}-ln\;w_{1}}{t_{2}-t_{1}}$$

where w is the dry mass of a sample collected at a determined day and t is the time expressed in days. This growth rate was calculated between day 1 and 7, as well as between day 7 and 14 post-inoculation.

### 4.7. DNA Extraction and Quantitative PCR

Plants roots, stems and leaves were sampled and stored at −80 °C. Each sample was freeze-dried and ground using 5 mm stainless steel beads and a MM 400 mixer mill (Retsch) until a fine powder was obtained. Genomic DNA was extracted from samples (30 mg dry weight) using the E.Z.N.A.^®^ Plant DNA Kit (Omega, Bio-tek (Norcross, GA, USA)), according to the manufacturer’s protocol. DNA from control plants and *Fusarium oxysporum* was extracted and quantified using a Quant-iT™ PicoGreen^®^ dsDNA quantification kit (Thermo Fischer Scientific, Waltham, MA, USA), according to the manufacturer’s protocol, in order to create standard scales for in planta fungi DNA quantification. To detect and quantify *L. usitatissimum* and *Fusarium oxysporum* genomic DNA, specific primers were used. For flax, the reference gene was chosen according to Fenart et al., 2010 (amplicon s_c3168, Forward primer: GACTCGTTCCTGAGGTCTGC, reverse primer: CCATCACACCCACAGTTCAG). For *Fusarium*, EF1α was used as the reference gene (Christopher Wattier, personal communication). qPCR was performed using a QuantStudio^TM^ 7 Flex System (Thermo Fisher Scientific) with PowerUp™ SYBR™ Green (Applied Biosystems, Waltham, MA, USA) reagent in 384 well microplates.

### 4.8. GC-MS Samples Extraction and Analysis

The polar primary metabolites were profiled using the method described by Pontarin et al. (2020) [32]. Between 10 and 15 mg of ground freeze-dried leaf was extracted 4 times using 400 µL of water/methanol (1:1) containing 200 nmol of ribitol, which was used as an internal standard. For each sample, 100 µL of extract was dried and derivatized. Thus, 40 µL of a pyridine solution containing 20 mg/mL of methoxyamine was added to the extract. The reaction was carried out for 2 h at 37 °C. Afterwards, 70 µL of MSTFA was added to each of the samples, which were then heated at 37 °C for 30 min. Following 1 h at ambient temperature, the samples were separated on a TRACE 1300 Gas Chromatograph and analyzed in a ISQ 7000 Single Quadrupole Mass Spectrometer. Each metabolite identity was verified using the standard or putatively by fragment matching with databases (putatively identified compounds are in brackets). All data were processed and recovered using the Chromeleon 7 software.

### 4.9. Ion Chromatography

The same sample extracts used for GC–MS were analyzed in a DIONEX ICS-900 Ion Chromatography system in the anion and cation modes, as previously described by Quéro et al. (2014).

### 4.10. PCA and Data Treatment

Principal component analysis (PCA) was realized with help from SIMCA-P software (v. 16.0, Umetrics, Umeå, Sweden). The Unit-Variance (UV) scaling method was used. Data curves and histograms were made in Excel (Office 16) software. Mann–Whitney tests were performed with the help of Vanted (V2.8.2) software.

## 5. Conclusions

Our metabolic and phenotypic analyses provide a general picture of the impact of *Fusarium oxysporum* on a resistant flax cultivar compared to a susceptible one. Both cultivars show phenotypic symptoms and a modulation of their primary metabolites profiles. The susceptible cultivar shows strong symptoms appearing from 1 week after the inoculation of the pathogen, comprising a decrease in the energetic metabolism, and a loss of water and nutrient absorption. On the other hand, the modifications in the primary metabolism, although close to those specific to abiotic stresses [33], do not seem completely related to osmotic stress, as suggested by the GABA and proline levels [30].

Our data highlight the presence of some markers, such as aromatic amino acids, (e.g., phenylalanine or tryptophan), that could explain Justess resistance. Some of these molecules, are directly involved in secondary metabolite pathways, such as phenylpropanoids pathway and are known to be induced during *Fusarium* infection in flax [34,35].

The conservation of a low pathogen quantity in the roots of Justess is probably key to the resistance strategy of this cultivar. Indeed, this could lead to less *Fusarium oxysporum* entering into the stem xylem, allowing a normal sap flow towards the aerial parts. This most likely explains the weaker symptoms in the aerial parts of Justess, while maintaining photosynthetic activity, a very green color and normal water consumption.

It is not clear whether the implementation of a physical barrier by Justess explains the big difference in resistance between the two cultivars; this is due to the similar penetration of the pathogen up to 7 DIA shown by our PCR data, even if clues of a parietal reconfiguration are observed in Progress. However, a chemical defense led by metabolites such as phytoalexins or phytoanticipins could participate in safeguarding the aerial part [18,19].

Finally, our study identified, for the first-time, different factors that are likely to play a role in the implementation of flax resistance to *Fusarium oxysporum*. Further work will be necessary to identify the relationship between these different factors and to decipher the pathways involved in the resistance in flax.

## Data Availability

Not applicable.

## Supplementary Materials

The following supporting information can be downloaded at: https://www.mdpi.com/article/10.3390/plants12101963/s1, Figure S1: Side views of Justess (resistant cultivar) and Progress (susceptible cultivar) plants in control (Ctrl) and infected (Inf) conditions at 14 days after inoculation; Figure S2: Analysis of side pictures of infected and non-infected flax with the PlantScreen™ system during 14 days of infection. Maximum quantum yield of photosystems (a,b) and evolution of plant color proportion for three groups of color (c–h) in Justess (resistant) and Progress (sensitive) varieties; Figure S3: Box plot representing the relative quantity of compounds of interest, contributing the most in global metabolic variations in plants leaves at 2, 4, 7 and 14 days after inoculation. Relative area is determined by an internal standard (ribitol). Error bars represent standard deviation of dataset per condition (n = 8). Significative difference between control and infected plants are indicated by stars. Mann–Whitney test (* for *p* < 0.05, ** for *p* < 0.01 and *** for *p* < 0.001).

## References

[1] Helbaek H. (1968). Plant Collecting, Dry-Farming, and Irrigation Agriculture in Prehistoric Deh Luran, Memoirs of the Museum of Anthropology.

[2] Zeist V. (1972). Paleobotanical results of the 1970 season at Cayonu, Turkey. Helinium.

[3] Łukaszewicz M., Szopa J., Krasowska A. (2004). Susceptibility of lipids from different flax cultivars to peroxidation and its lowering by added antioxidants. Food Chem..

[4] Xie Y., Gan Y., Li Y., Niu J., Gao Y., An H., Li A. (2015). Effect of Nitrogen Fertilizer on Nitrogen Accumulation, Translocation, and Use Efficiency in Dryland Oilseed Flax. Agron. J..

[5] Mazur W., Fotsis T., Wähälä K., Ojala S., Salakka A., Adlercreutz H. (1996). Isotope Dilution Gas Chromatographic–Mass Spectrometric Method for the Determination of Isoflavonoids, Coumestrol, and Lignans in Food Samples. Anal. Biochem..

[6] Subedi K., Yu H.M., Newell M., Weselake R.J., Meesapyodsuk D., Qiu X., Shah S., Field C.J. (2015). Stearidonic acid-enriched flax oil reduces the growth of human breast cancer in vitro and in vivo. Breast Cancer Res. Treat.

[7] Deshpande R., Raina P., Shinde K., Mansara P., Karandikar M. (2019). Flax seed oil reduced tumor growth, modulated immune responses and decreased HPV E6 and E7 oncoprotein expression in a murine model of ectopic cervical cancer. Prostaglandins Other Lipid Mediat..

[8] Gao Y. (2020). Oilseed flax (*Linum usitatissimum* L.), an emerging functional cash crop of China. Oil Crop Sci..

[9] Kostyn K., Czemplik M., Kulma A., Bortniczuk M., Skała J., Szopa J. (2012). Genes of phenylpropanoid pathway are activated in early response to *Fusarium* attack in flax plants. Plant Sci..

[10] Kang Z., Buchenauer H. (2002). Studies on the Infection Process of *Fusarium Culmorum* in Wheat Spikes: Degradation of Host Cell Wall Components and Localization of Trichothecene Toxins in Infected Tissue. Eur. J. Plant Pathol..

[11] Srivastava S., Pathak N., Srivastava P. (2011). Identification of limiting factors for the optimum growth of *Fusarium oxysporum* in liquid medium. Toxicol. Int..

[12] Olivain C., Trouvelot S., Binet M.N., Cordier C., Pugin A., Alabouvette C. (2003). Colonization of Flax Roots and Early Physiological Responses of Flax Cells Inoculated with Pathogenic and Nonpathogenic Strains of *Fusarium oxysporum*. Appl. Environ. Microbiol..

[13] Michielse C.B., Rep M. (2009). Pathogen profile update: *Fusarium oxysporum*. Mol. Plant Pathol..

[14] Wojtasik W., Kulma A., Kostyn K., Szopa J. (2011). The changes in pectin metabolism in flax infected with *Fusarium*. Plant Physiol. Biochem..

[15] Wojtasik W., Kulma A., Namysł K., Preisner M., Szopa J. (2015). Polyamine metabolism in flax in response to treatment with pathogenic and non-pathogenic *Fusarium* strains. Front. Plant Sci..

[16] Ohara T., Tsuge T. (2004). FoSTUA, Encoding a Basic Helix-Loop-Helix Protein, Differentially Regulates Development of Three Kinds of Asexual Spores, Macroconidia, Microconidia, and Chlamydospores, in the Fungal Plant Pathogen *Fusarium oxysporum*. Eukaryot. Cell.

[17] Burketova L., Trda L., Ott P.G., Valentova O. (2015). Bio-based resistance inducers for sustainable plant protection against pathogens. Biotechnol. Adv..

[18] Paxton J.D. (1981). Phytoalexins–A working Redefinition. Phytopathology.

[19] VanEtten H.D., Mansfield J.W., Bailey J.A., Farmer E.E. (1994). Two Classes of Plant Antibiotics: Phytoalexins versus “Phytoanticipins”. Plant Cell.

[20] Hunt R., Thomas B., Murray B.G., Murphy D.J. (2017). Growth Analysis, Individual Plants.

[21] Zuk M., Szperlik J., Hnitecka A., Szopa J. (2019). Temporal biosynthesis of flavone constituents in flax growth stages. Physiol. Biochem..

[22] Genty B., Briantais J.M., Baker N.R. (1989). The relationship between the quantum yield of photosynthetic electron transport and quenching of chlorophyll fluorescence. Biochem. Biophys. Acta Gen. Subj..

[23] Chen C.Y., Chen S.Y., Liu C.W., Wu D.H., Kuo C.C., Lin C.C., Chou H.P., Wang Y.Y., Tsai Y.C., Lai M.H. (2020). Invasion and Colonization Pattern of *Fusarium fujikuroi* in Rice. Phytopathology.

[24] Bolton M.D. (2009). Primary metabolism and plant defense-fuel for the fire. Mol. Plant Microbe Interact..

[25] White A.C., Rogers A., Rees M., Osborne C.P. (2016). How can we make plants grow faster?. A source–sink perspective on growth rate. J. Exp. Bot..

[26] Smith A.M., Stitt M. (2007). Coordination of carbon supply and plant growth. Plant Cell Environ..

[27] Isayenkov S., Isner J.C., Maathuis F.J. (2010). Vacuolar ion channels: Roles in plant nutrition and signalling. FEBS Lett..

[28] Vijayakumari K., Jisha K.C., Puthur J.T. (2016). GABA/BABA priming: A means for enhancing abiotic stress tolerance potential of plants with less energy investments on defence cache. Acta Physiol. Plant.

[29] Shelp B.J., Aghdam M.S., Flaherty E.J. (2021). γ-Aminobutyrate (GABA) Regulated Plant Defense: Mechanisms and Opportunities. Plants.

[30] Quéro A., Molinié R., Elboutachfaiti R., Petit E., Pau-Roblot C., Guillot X., Mesnard F., Courtois J. (2014). Osmotic stress alters the balance between organic and inorganic solutes in flax (*Linum usitatissimum*). J. Plant Physiol..

[31] Bai G., Jenkins S., Yuan W., Graef G.L., Ge Y. (2018). Field-Based Scoring of Soybean Iron Deficiency Chlorosis Using RGB Imaging and Statistical Learning. Front. Plant Sci..

[32] Pontarin N., Molinié R., Mathiron D., Tchoumtchoua J., Bassard S., Gagneul D., Thiombiano B., Demailly H., Fontaine J.X., Guillot X. (2020). Age-Dependent Metabolic Profiles Unravel the Metabolic Relationships within and between Flax Leaves (*Linum usitatissimum*). Metabolites.

[33] Fàbregas N., Fernie A.R. (2019). The metabolic response to drought. J. Exp. Bot..

[34] Boba A., Kostyn K., Kostyn A., Wojtasik W., Dziadas M., Preisner M., Szopa J., Kulma A. (2017). Methyl Salicylate Level Increase in Flax after *Fusarium oxysporum* Infection Is Associated with Phenylpropanoid Pathway Activation. Front. Plant Sci..

[35] Galindo-González L., Deyholos M.K. (2016). RNA-seq Transcriptome Response of Flax (*Linum usitatissimum* L.) to the Pathogenic Fungus *Fusarium oxysporum* f. sp. *lini*. Front. Plant Sci..

