# Supplementary material for: Pathogen Stopping and Metabolism Modulation Are Key Points to Linum usitatissimum L. Early Response against Fusarium oxysporum
Source: Plants (Basel). 2023 May 12;12(10):1963. doi: 10.3390/plants12101963 (PMC10223704; doi:10.3390/plants12101963)
Supplement: Supplementary file 1 [file plants-12-01963-s001.zip › plants-2289114-supplementary.pdf]

Supplementary Materials

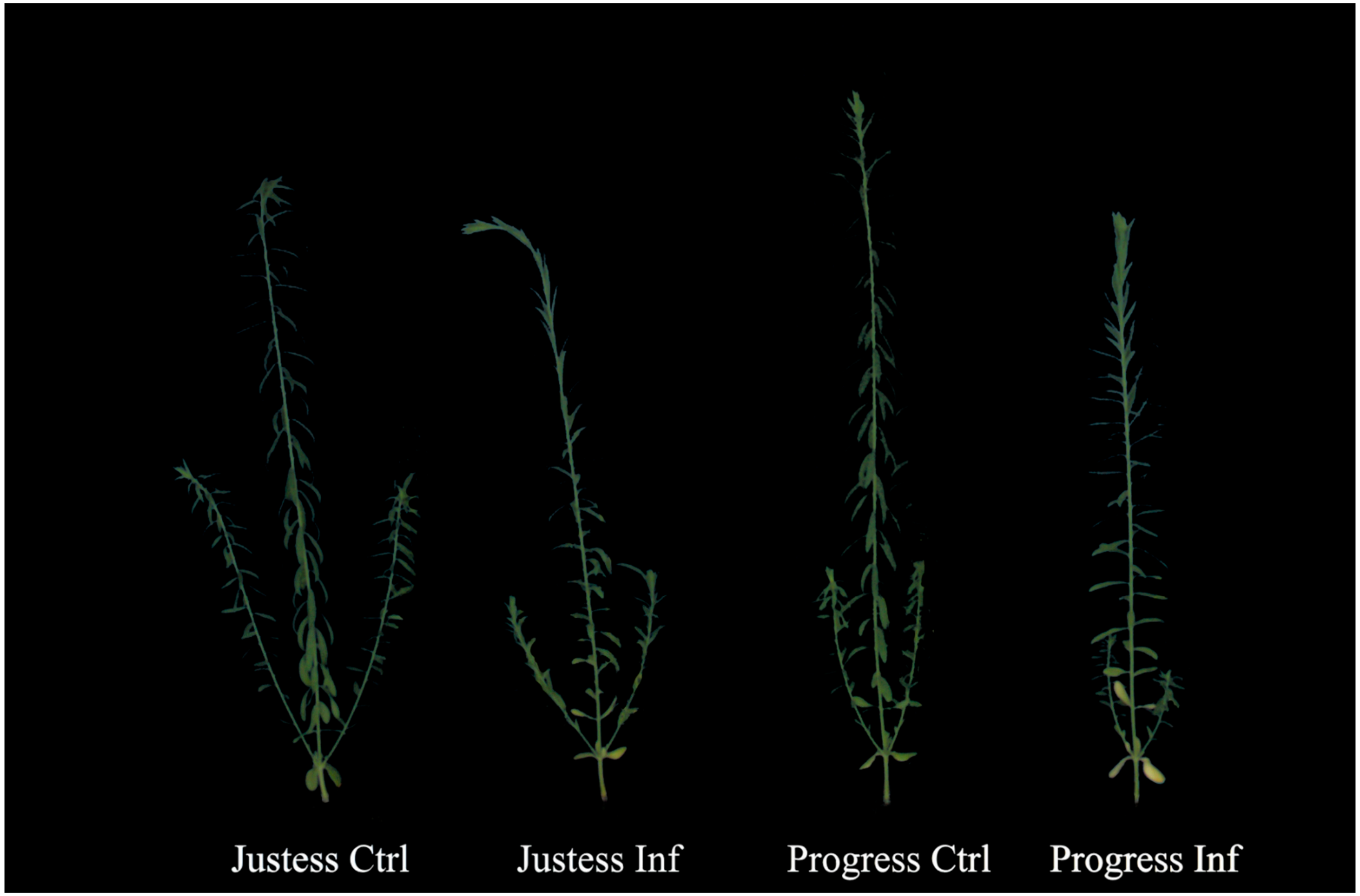

Figure S1. Side views of Justess (resistant variety) and Progress (sensitive variety) plants in control (Ctrl) and infected (Inf) conditions at 14 days after inoculation.

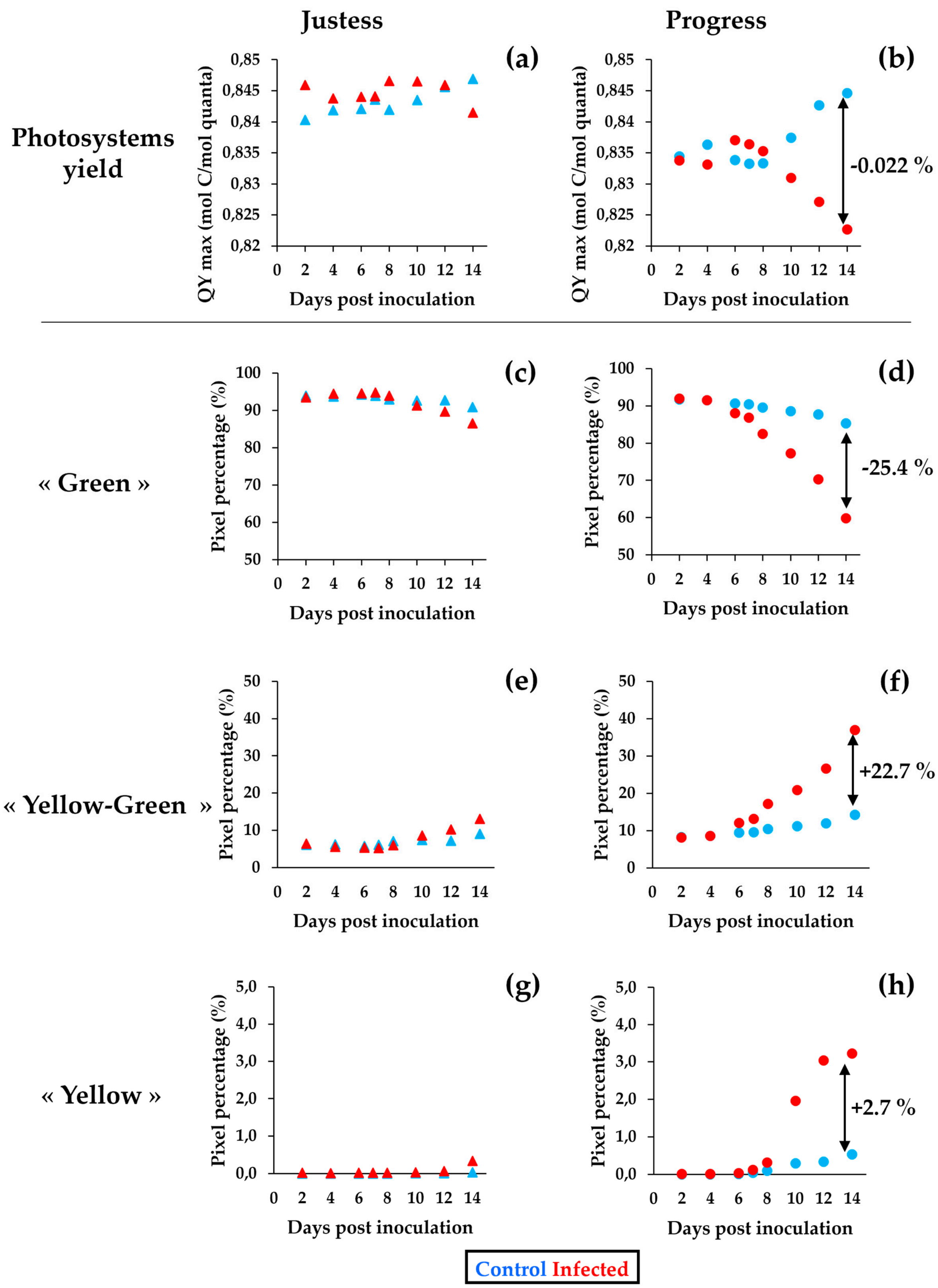

Figure S2. Analysis of side pictures of infected and non-infected flax with the PlantScreen™ system during 14 days of infection. Maximum quantum yield of photosystems (**a-b**) and evolution of plant color proportion for 3 groups of color (**c-h**) in Justess (resistant) and Progress (sensitive) varieties.

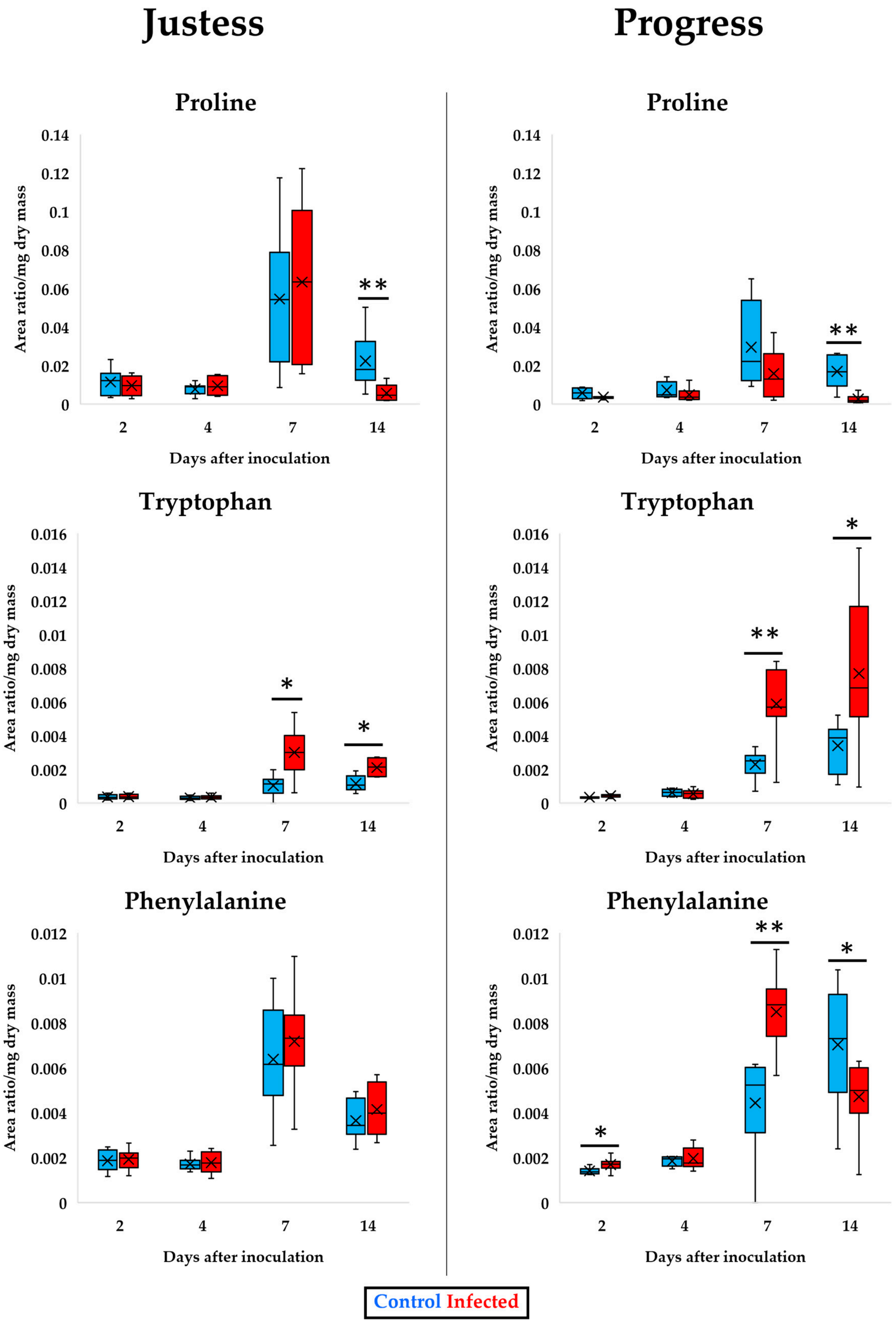
Justess
Progress
Proline
Tryptophan
Phenylalanine
Area ratio/mg dry mass
0.14
0.12
0.1
0.08
0.06
0.04
0.02
0
0.016
0.014
0.012
0.01
0.008
0.006
0.004
0.002
2
4
7
14
Days after inoculation
**
*
Control Infected

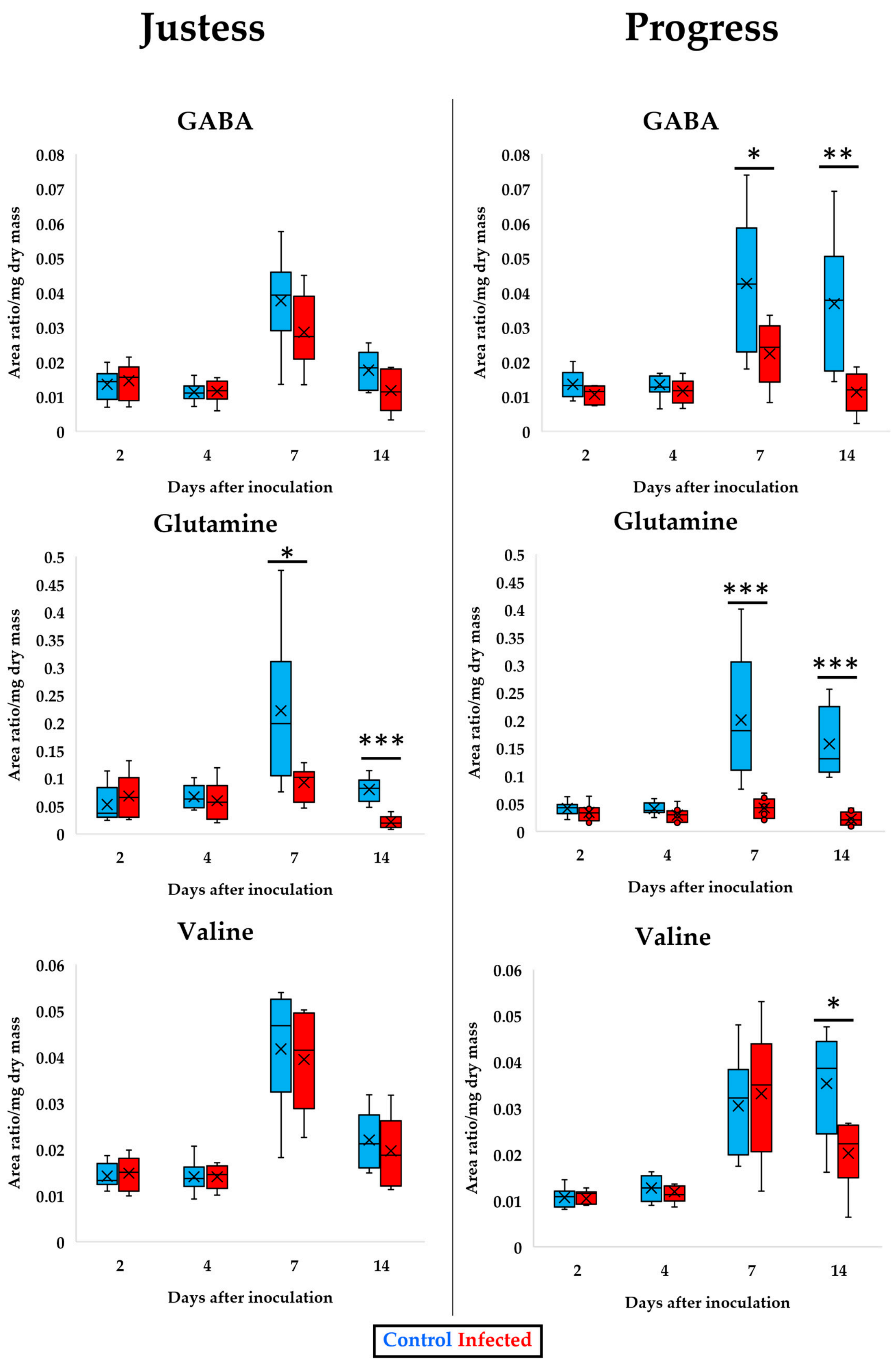
Justess
Progress
GABA
Glutamine
Valine
Area ratio/mg dry mass
Days after inoculation
2
4
7
14
0
0.01
0.02
0.03
0.04
0.05
0.06
0.07
0.08
0.05
0.1
0.15
0.2
0.25
0.3
0.35
0.4
0.45
0.5
*
**
***
Control Infected

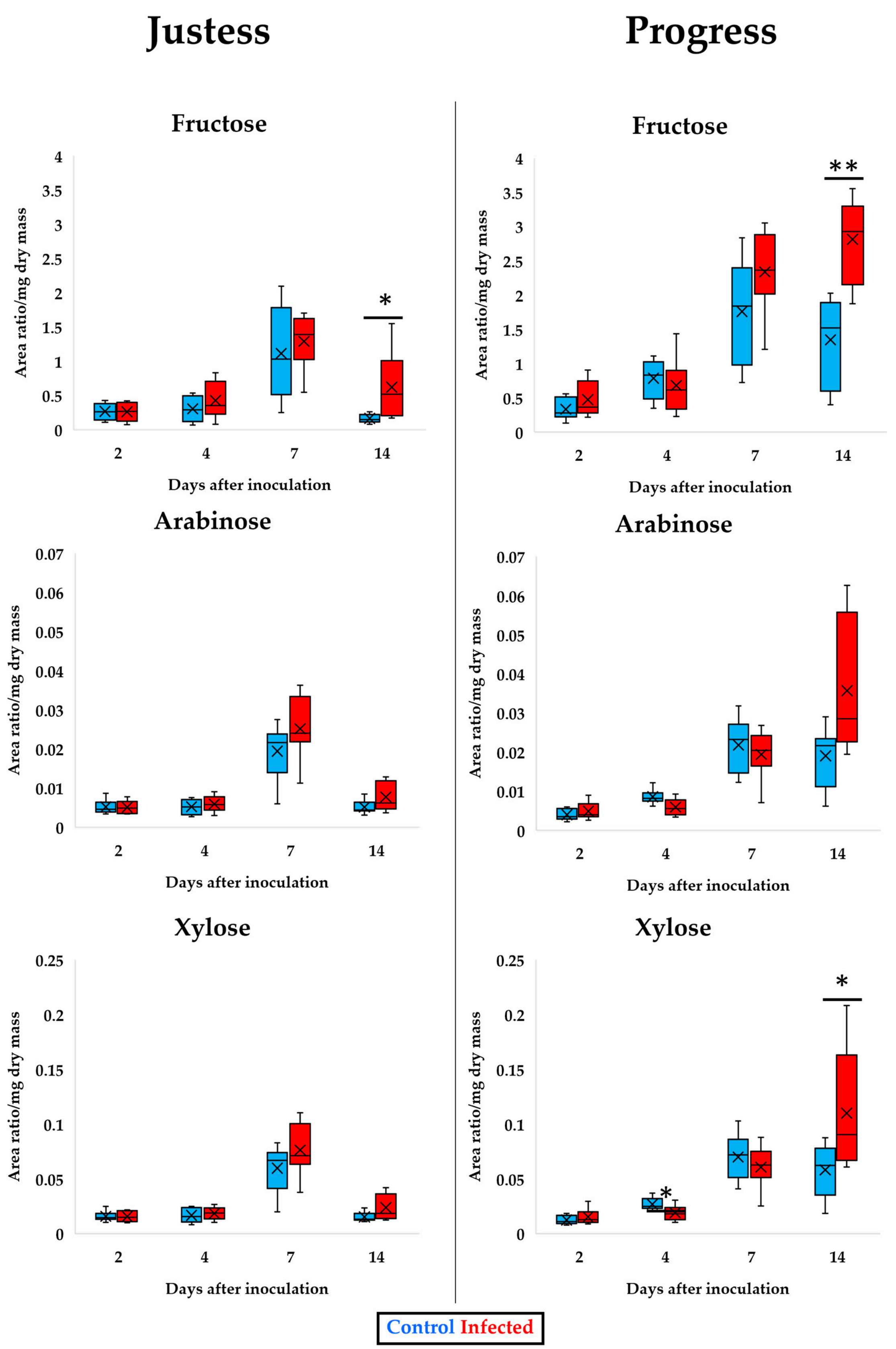

Justess
Progress
Fructose
Area ratio/mg dry mass
Days after inoculation
Arabinose
Xylose
Control Infected

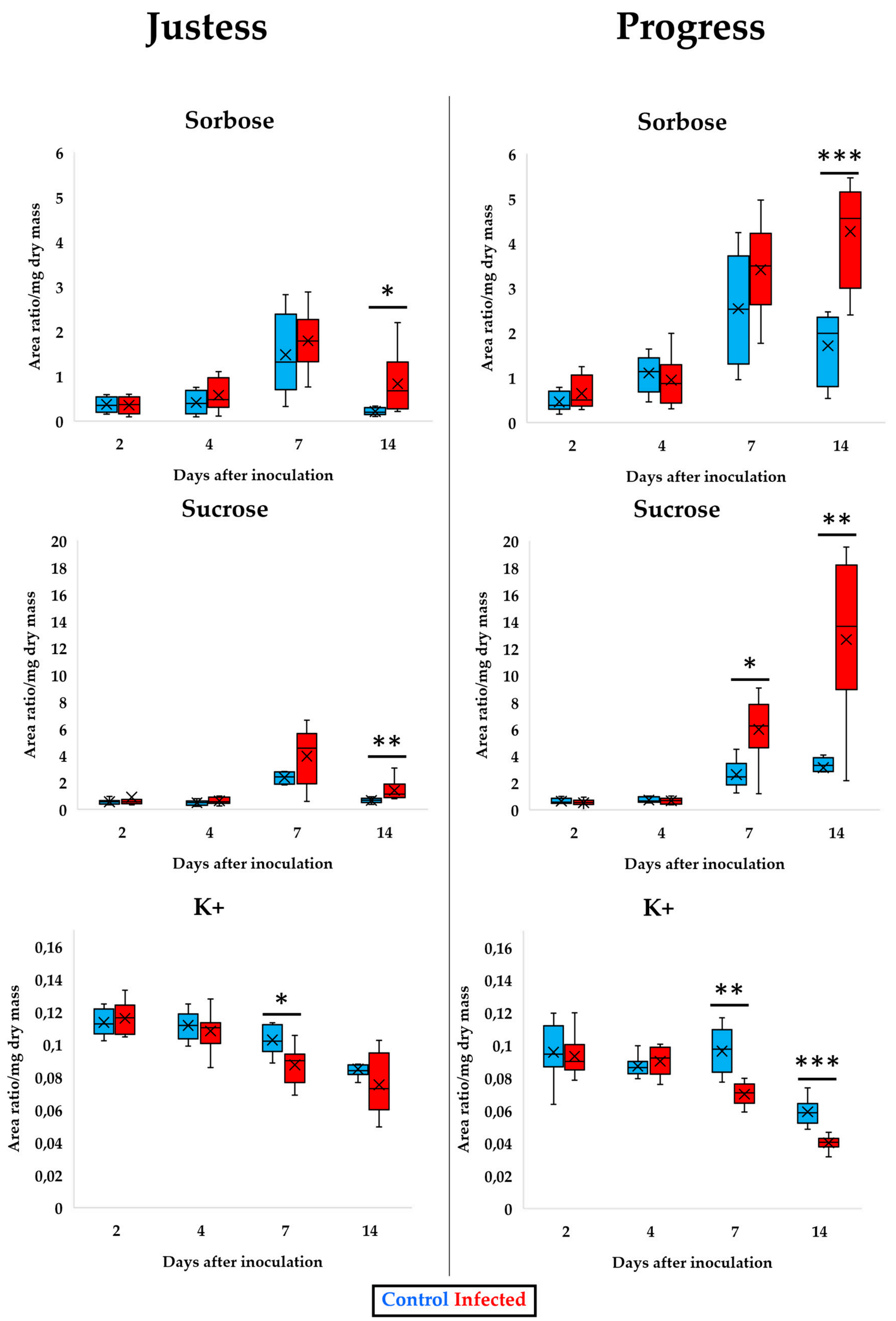

Figure S3. Box plot representing the relative quantity of compounds of interest, contributing the most in global metabolic variations, in plants leaves at 2-, 4-, 7- and 14-days after inoculation. Relative area is determined by an internal standard (ribitol). Error bars represent standard deviation of data set per condition (n=8). Significative difference between control and infected plants are indicated by stars. Mann-Whitney test (* for p<0.05, ** for p<0.01 and *** for p<0.001).
